# Comparative efficacy of nonpharmaceutical therapy in the treatment of dysphagia after stroke: A protocol for systematic review

**DOI:** 10.1097/MD.0000000000019115

**Published:** 2020-02-28

**Authors:** Weixun Qin, Zhijie Wang, Yue Zhong, Qing Yuan, Xin Jiang, Jing Gao, Junyan Wu, Yu Zhang

**Affiliations:** a Clinical Medical College of Acupuncture and Rehabilitation, Guangzhou University of Chinese Medicine, Guangzhou; b Shanxi Province Hospital of Traditional Chinese Medical, Taiyuan, China.

**Keywords:** dysphagia, network meta-analysis, nonpharmacological, post-stroke, protocol, systematic review

## Abstract

**Background:**

Dysphagia is one of the major post-stroke complications that can severely damage a patient's quality of life. An increasing number of studies have demonstrated that many kinds of nonpharmacological treatments can be used for post-stroke dysphagia. However, there is not enough evidence evaluating the effectiveness and safety of these interventions. This study will conduct a systematic review, and Bayesian network meta-analysis, of nonpharmacological treatments in order to provide evidence for a future study investigating more options for post-stroke dysphagia.

**Methods:**

Randomized controlled trials (RCTs) of adult patients aged >18 years old who meet the criteria for a diagnosis of post-stroke dysphagia will be included, regardless of gender, nationality, or education level. Four Chinese databases (CNKI, SinoMed, Wanfang Database, and the Chinese Scientific Journal Database) and four English databases (Web of Science, MEDLINE, Embase, and the Cochrane Library) will be searched. Two independent reviewers will evaluate the title summary for each RCT. Disagreements will be discussed with a third commentator. Standard pairwise meta-analysis, including heterogeneity analysis, subgroup analysis, and sensitivity analysis, will be performed using the RevMan 5.3 software, and the risk of bias assessment will be conducted based on the methodological quality of the included trials recommended by the Cochrane Handbook 5.1. The Bayesian network meta-analysis will be performed using R-3.3.2 software. The quality evaluation of this study will be completed using the World Health Organization's Grading of Recommendations, Assessment, Development, and Evaluation.

**Results:**

This study will summarize all the selected trials aimed at estimating the effectiveness, as well as safety, of applying nonpharmacological treatments to post-stroke dysphagia.

**Conclusion:**

This systematic review will provide evidence to assess the validity and safety of applying different types of nonpharmacological treatments for post-stroke dysphagia, which may provide clinicians with more choices in the treatment of this disease.

**PROSPERO registration number:**

CRD42019119368.

## 1 Introduction

Dysphagia (a difficulty in, or discomfort when, swallowing) is 1 of the major poststroke complications. The incidence rate of poststroke swallowing disorders is as high as 37% to 78% (depending on patient characteristics, research design, the type and severity of the stroke, assessment time, and diagnostic methods), which becomes an independent quality-of-life risk factor that seriously affects the prognosis of stroke patients.^[1]^ Reports indicate that patients with dysphagia have an 8.5-fold higher risk of death when compared with those who can swallow normally.^[2]^ Because of dysphagia, food and water are mistakenly inhaled into the lungs, which may cause lung infections and aspiration pneumonia. Dysphagia may also cause an inadequate intake of nutrients and water, which can lead to malnutrition, electrolyte disorders, aspiration pneumonia, and even apnea.^[3]^ Dysphagia also affects the patients’ quality of life and mental health, disrupting their daily lives and causing a social burden.^[4]^

The current treatment methods^[5]^ for dysphagia include compensation therapy (the adjustment of eating postures, a change in food characteristics, and the adjustment of eating tools), swallowing function training (oral sensory training and swallowing muscle training), physiotherapy (various therapeutic methods including electrical, magnetic, cold, and thermal stimulation), alternative therapy (acupuncture, psychotherapy, transcranial magnetic stimulation [rTMS], and balloon dilation), and drugs. Although drugs improve swallowing speed, pharmacotherapy has only a limited amount to offer patients with more severe complications. At the same time, pharmacotherapy requires a swallowing movement that cooperates with the patient's tongue, which is difficult for patients with a poor swallowing function. Some randomized controlled trials (RCTs) have suggested that nonpharmacological therapy may improve the remaining dysphagia experienced by patients after having a stroke. Noninvasive cortical stimulation^[5–9]^ (such as transcranial direct current stimulation [tDCS] and rTMS), electrical stimulation, exercise, acupuncture, and some other alternative therapies^[10–18]^ may potentially be viable alternative treatments for patients with poststroke dysphagia. These nonpharmacological therapies have no reported side effects or drug interactions according to the current evidence, so they are more easily accepted by patients than pharmacological treatments are. However, most current meta-analyses have only compared the effectiveness of 2 nonpharmacological therapies.^[19–21]^ To evaluate the effect of different types of nonpharmacological therapies on patients with poststroke dysphagia, it is necessary to carry out a network meta-analysis to draw a convincing conclusion.

This systematic review and network meta-analysis will aim to solve this problem, and evaluate the potential availability and safety of all the different types of nonpharmacological interventions for poststroke dysphagia.

### 1.1 Objective

The objective of this study will be to compare the effect of different nonpharmacological interventions for poststroke dysphagia using a network meta-analysis of RCTs.

## 2 Methods

### 2.1 Design

A systematic review and network meta-analysis will be carried out in this study, which will only include RCTs and will exclude equivalence trials and clinical inferiority trials.

### 2.2 Registration

This study is consistent with the Preferred Reporting Items for Systematic Reviews and Meta-analyses (PRISMA) statements. We have registered the protocol on the International Prospective Register of Systematic Reviews (PROSPERO) (registration number: CRD42019119368).

### 2.3 Eligibility criteria

#### 2.3.1 Types of studies

This study will only include RCTs, and the study design will strictly follow P-Population, I-Intervention, C-Control, O-Outcome, S-Study designs (PICOS) principles. Any RCTs of nonpharmacological treatment for poststroke dysphagia will be included in the study; studies using pharmacological treatment will not be included. In addition, we will not admit non-RCTs, animal experiments, human cell or tissue experiments, or repeated published studies.

#### 2.3.2 Types of participants

Adults aged >18 years who meet the criteria for a diagnosis of poststroke dysphagia according to their National Institutes of Health Stroke Scale score and a Video Fluoroscopic Swallowing Study (VFSS)^[6]^ will be included, regardless of sex, nationality, or education level. Patients with no history of other neurological diseases or with pre-existing oral and maxillofacial surgery that involved the lips and/or tongue will be involved. Participants who do not meet the diagnostic criteria, or who are on medication, will be excluded.

#### 2.3.3 Types of interventions

The treatment group will include only nonpharmacological intervention studies, including (but not limited to) complementary and alternative therapies such as acupuncture, massage, cupping, and other methods used to stimulate acupoints; surface neuromuscular electrical stimulation such as tDCS, transcranial magnetic stimulation (rTMS), and surface neuromuscular electrical stimulation (sNMES); rehabilitation training such as tongue to jaw resistance training, tongue training, and swallowing training; and psychological interventions.

The control group will use a variety of different conventional treatments such as placebo intervention, no treatment, false stimulation under the same conditions, and usual care.

#### 2.3.4 Types of outcomes

Primary outcome indicators will include changes in swallowing function, determined by the following: a VFSS (this refers to the special deglutigraphy in the swallowing movement of the pharynx, mouth, larynx, and esophagus under x-ray fluoroscopy, which is regarded as the ideal method, and also the gold standard, for the examination of dysphagia); and a functional dysphagia scale score (a total of 11 movements including lipclosure score, bolus formation, residue in oral cavity, oral transit time, triggering of pharyngeal swallow, laryngeal elevation and epiglottic closure, nasal penetration, residue in valleculae, coating of pharyngeal wall after swallow, and pharyngeal transit time, which are scored “0” to “100” points; 0 points indicate a normal swallowing function, whereas 100 points indicate a severely impaired swallowing function).

Secondary outcome indicators will include a tongue pressure assessment, a quality of life related to the swallowing questionnaire, a the Rosenbek Penetration-Aspiration Scale (PSA) scale, a Dysphagia Outcome and Severity Scale (DOSS) scale, OTT (oral running time), PTT (pharyngeal transit time), and TTT (total running time = OTT + PTT).

### 2.4 Information sources

We will search electronic databases established before November, 2019, including 4 Chinese databases (CNKI, SinoMed, Wanfang Database, and the Chinese Scientific Journal Database [VIP]) and 4 English databases (Web of Science, MEDLINE, Embase, and the Cochrane Library). In addition, gray references such as conference papers and bibliographies will also be included.

### 2.5 Search strategy

**Figure 1:**
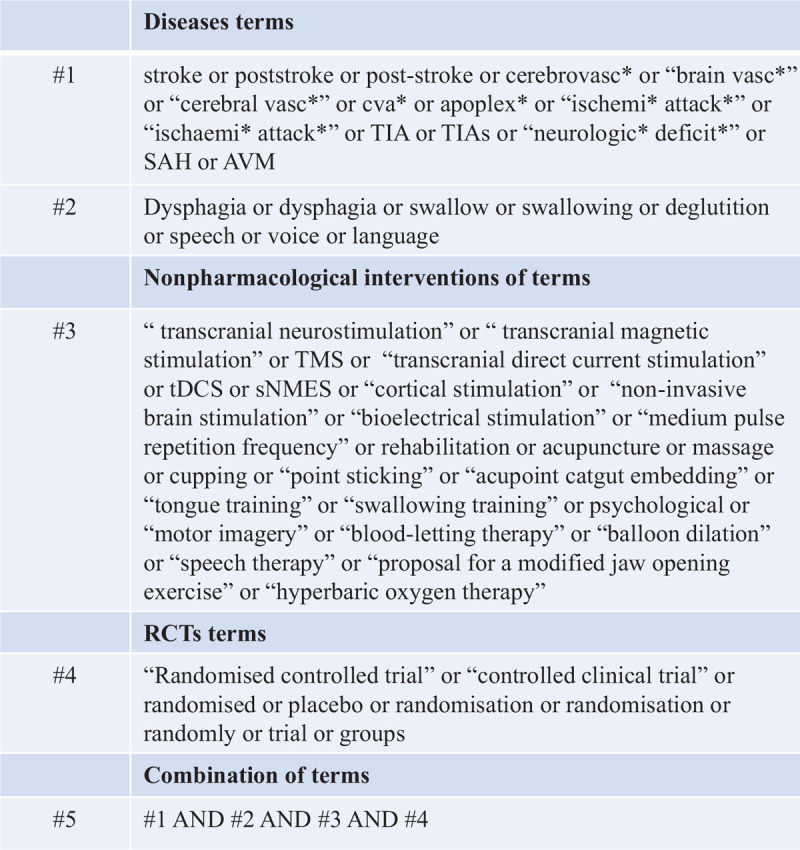
Search strategy: PubMed/MEDLINE.

A comprehensive search strategy will be developed for PubMed or MEDLINE (Fig. 1).

### 2.6 Data collection and management

#### 2.6.1 Selection of studies

**Figure 2:**
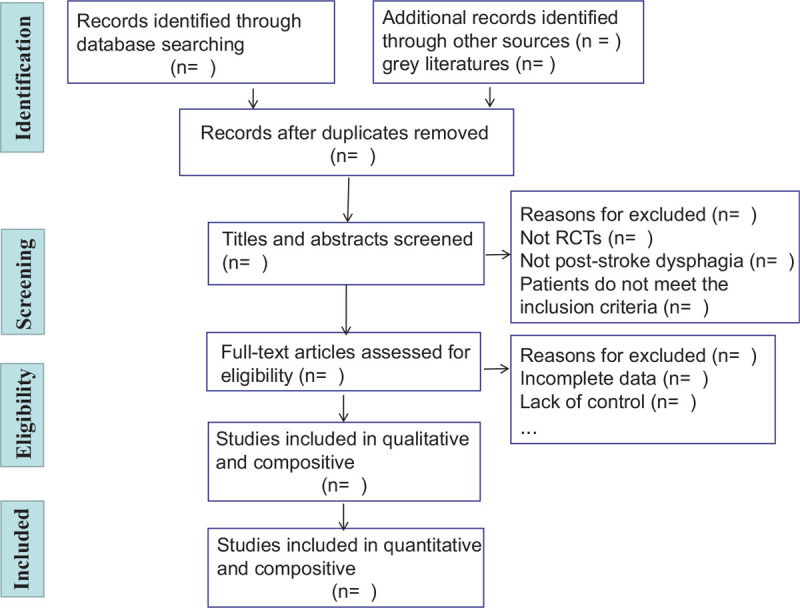
Preferred Reporting Items for Systematic Reviews and Meta-Analysis (PRISMA) flow chart of the study process.

The evaluation staff will be trained, and the literature used will be prescreened, to ensure the standardization of the literature screening process. The selection process requires at least 2 independent evaluators (W.Z.J. and Z.Y.), both of whom are masters of (non)acupuncture and moxibustion. Two reviewers will independently screen the titles and abstracts to select eligible articles, and, after completing the screening phase, articles that are deemed relevant by at least 1 of the reviewers will be subjected to a full-text review. Any disagreements between the 2 reviewers will be resolved by discussion with a third reviewer (J.X.). The study selection process will be detailed in the systematic evaluation plan and the full text. The following steps are included (Fig. 2): select the studies and exclude any duplications with EndNote; read the title and abstract of each study to exclude unrelated studies that clearly do not meet the inclusion criteria; and analyze and determine any duplicated publications.

#### 2.6.2 Data extraction

The data will be extracted by 2 independent reviewers (W.Z.J. and Z.Y.).

The extracted information will include study identification (first author, publication year, publication type, publication region, and the publishing journal/magazine), study characteristics (study setting, study design, study inclusion criteria, and the inclusion criteria for poststroke dysphagia), inclusion criteria (age, onset time, sex, imaging examination, and the degree of swallowing dysfunction), intervention type (such as acupuncture, rTMS, tDCS, or rehabilitation training), and other confounding factors may lead to inaccuracy (funding sources, key conclusions drawn by the authors, the evaluation of confounders, references to other studies, etc).

### 2.7 Assessment risk of bias and reporting of study quality

We will use the Cochrane Collaboration's tool for assessing the risk of bias (RoB2)^[19]^ in the methodological quality of the RCTs. It includes the following 7 aspects: random sequence generation (selection bias); allocation concealment (selection bias); blinding of participants and personnel (performance bias); blinding of outcome assessment (detection bias); incomplete outcome data (attrition bias); selective reporting (reporting bias); and other biases.

The risk of bias will be classified as high, medium, or low. All the differences will be discussed by 2 reviewers, or, if not, will be discussed with the third reviewer.

### 2.8 Comprehensive statistical analysis of data

#### 2.8.1 Standard pair-wise meta-analysis

Data analysis and pair-wise meta-analysis will be performed using the RevMan 5.3 software through the Cochrane Collaboration. We will determine the effect size based on the form and type of the study. Relative risk and standardized mean difference have been selected as the effect size expressions for dichotomous and continuous variables, with 95% confidence intervals (95% CIs). We will use Cochrane *Q* and *P* values to qualitatively analyze the statistical heterogeneity of the results. The larger the *Q* value relative to the degree of freedom (d.f.) and the smaller the *P* value, the greater the possibility of heterogeneity. When the *P* value is <.05, it will indicate that there is statistical heterogeneity between the groups. We will conduct a quantitative analysis of the heterogeneity of the results through *I*^2^. When *I*^2^ < 25%, it means the heterogeneity is low. When 25% < *I*^2^ < 50%, it indicates a moderate degree of heterogeneity in the study. *I*^2^ > 75% means the heterogeneity is large. In general, when *I*^2^ > 50%, it indicates that there is substantial heterogeneity.^[22]^ If there is heterogeneity between the results, we will use a subgroup analysis.^[23]^ Meta-regression will be conducted if there are more than 2 variables. A sensitivity analysis will be performed to determine data reliability based on missing data, sample size, and heterogeneity. When the data analysis meets the homogeneity, a fixed-effect model can be used. If there is no homogeneity, a random-effect model will be used to estimate the combined effect.^[24]^ We will use a funnel plot and Nfs to estimate the impact of publication bias on the results of the meta-analysis.^[22]^

#### 2.8.2 Network meta-analysis

We will use the “gemtc” V.0.8.1 package of R-3.3.2 software for network meta-analysis based on a Bayesian framework.^[25]^ Parameters such as mean and standard deviation (SD) will use noninformative prior distributions and normal prior distributions. A sensitivity analysis will also be used to evaluate the effect of different prior information on the results. Each Markov chain Monte Carlo will run 100,000 times, and the total number of iterations will be 300,000. The first 50,000 times will be discarded as the number of simulated annealings. The convergence evaluation will use Brooks–Gelman–Rubin plots, and we will calculate the 95% CI. If there is a closed loop, the node analysis method will be used to detect the consistency,^[26]^ calculating the difference and Bayesian *P* value. A sensitivity analysis will be conducted to judge the stability of the results when any data are missing. This study method will be reported strictly based on the Preferred Reporting Items for Systematic Reviews and Meta-analysis Protocol (PRISMA-P).

#### 2.8.3 Grading of the quality of evidence

The quality evaluation of this study will be completed using the Grading of Recommendations, Assessment, Development, and Evaluations (GRADE) established by the the World Health Organization.^[27]^ Because the network meta-analysis is based on RCTs, the basic principles of GRADE's application in network meta-analysis will mainly examine 5 degrading factors: risk of bias, indirectness, inconsistency, imprecision, and publication bias.

## 3 Discussion

More and more studies are showing that poststroke dysphagia can be improved through some nonpharmacological treatments. During surface neuromuscular electrical treatment, a systematic review of 8 RCTs, with a total of 329 patients, reported tDCS seemed to be more effective than that what without neuromuscular electrical stimulation in the treatment of poststroke dysphagia, in the short term, and considering the limited number of studies available.^[28]^ Another systematic review of 6 RCTs, with a total of 163 patients, concluded that repetitive transcranial magnetic stimulation had a positive effect on poststroke dysphagia.^[29]^ In addition, 2 meta-analyses, with a total of 8200 participants, provided new evidence supporting the efficacy and safety of acupuncture therapy for poststroke dysphagia in the short term when compared with medication.^[30,31]^ However, this literature lacked evidence comparing the benefits of different nonpharmacological interventions for poststroke dysphagia. Our systematic review and network meta-analysis will comprehensively analyze all the evidence available regarding the effects of different nonpharmacological interventions for poststroke dysphagia. This is different to the traditional meta-analysis method in that a Bayesian network meta-analysis will provide comparisons of both direct and indirect interventions.

### 3.1 Ethics and dissemination

As this study does not involve raw data collection, no ethical review is required. The results of this study will provide strong evidence for the treatment of poststroke dysphagia using nonpharmacological treatments. This will help clinicians to make treatment decisions for poststroke dysphagia. This systematic review will be published in a peer-reviewed journal and at an international academic conference.

### 3.2 Strengths and limitations

This will be the first systematic review and network meta-analysis to synthesize the utility and safety of nonpharmacological treatments for improving poststroke dysphagia. It could provide a basis for clinicians to determine the benefits, and also drawbacks, of the various methods of treatment of poststroke dysphagia and provide evidence for the clinical treatment of dysphagia in the field of nonpharmacological therapy, improving the patients’ quality of life. The methods and quality assessments will follow systematic review guidelines and criteria to minimize the risk of bias. However, a potential limitation of this study could be that different types of nonpharmacological treatment may cause greater heterogeneity, and our conclusions will depend on the quality and quantity of the original studies.

## Author contributions

**Conceptualization:** Weixun Qin, Yu Zhang.

**Data extraction:** Xin Jiang, Jing Gao.

**Investigation:** Yue Zhong, Qing Yuan.

**Methodology:** Weixun Qin, Yu Zhang, Zhijie Wang

**Project administration:** Qing Yuan

**Resources:** Zhijie Wang, Jing Gao, Qing Yuan

**Software:** Yu Zhang, Zhijie Wang

**Writing – original draft:** Weixun Qin, Yu Zhang, Zhijie Wang

**Writing – review & editing:** Xin Jiang, Qing Yuan

Weixun Qin orcid: 0000-0001-8512-5686.
